# Malignant Keratitis Caused by a Highly-Resistant Strain of Fusarium Tonkinense from the *Fusarium Solani* Complex

**DOI:** 10.3390/jof7121093

**Published:** 2021-12-18

**Authors:** Jens Schrecker, Berthold Seitz, Tim Berger, Loay Daas, Wolfgang Behrens-Baumann, Claudia Auw-Hädrich, Sabine Schütt, Sabine Kerl, Sascha Rentner-Andres, Herbert Hof

**Affiliations:** 1 Department of Ophthalmology, Rudolf Virchow Klinikum Glauchau, Virchowstraße 18, D-08371 Glauchau, Germany; jens.schrecker@t-online.de; 2 Department of Ophthalmology, Saarland University Medical Center, Kirrbergstr. 100, D-66424 Homburg, Germany; berthold.seitz@uks.eu (B.S.); tim.berger@uks.eu (T.B.); loay.daas@uks.eu (L.D.); 3 Emeritus, Department of Ophthalmology, Otto-von-Guericke-University Magdeburg, Eckenbornweg 5j, D-37075 Göttingen, Germany; profbb@t-online.de; 4 Eye Center, Medical Center, Faculty of Medicine, University of Freiburg, Kilianstr. 5, D-79106 Freiburg im Breisgau, Germany; claudia.auw-haedrich@uniklinik-freiburg.de; 5 MVZ Labor Limbach and Colleagues, Im Breitspiel 16, D-69126 Heidelberg, Germany; sabine.schuett@labor-limbach.de (S.S.); sabine.kerl@labor-limbach.de (S.K.); 6 Limbach Analytics GmbH, Arotop Laboratories, Dekan-Laiststr. 9, D-55129 Mainz, Germany; s.rentner-andres@analytics-mainz.de

**Keywords:** *Fusarium solani*, ocular infection, fungal keratitis, keratoplasty, antimycotics

## Abstract

*Fusarium* spp. are moulds ubiquitously distributed in nature and only occasionally pathogenic for humans. Species of the *Fusarium solani* complex are the predominant keratitis-inducing pathogens, because they are endowed with proper virulence factors. These fungi can adhere to the cornea creating a biofilm and, with the help of enzymes and cytotoxins, penetrate the cornea. Whereas an intact cornea is hardly able to be invaded by *Fusarium* spp. in spite of appropriate virulence factors, these opportunistic fungi may profit from predisposing conditions, for example mechanical injuries. This can lead to a progressive course of corneal infection and may finally affect the whole eye up to the need for enucleation. Here, we present and discuss the clinical, microbiological and histopathological aspects of a particular case due to Fusarium tonkinense of the *Fusarium solani* complex with severe consequences in a patient without any obvious predisposing factors. A broad portfolio of antifungal agents was applied, both topically and systemically as well as two penetrating keratoplasties were performed. The exact determination of the etiologic agent of the fungal infection proved likewise to be very challenging.

## 1. Introduction

*Fusarium* spp. are a heterogenous group of ascomycetous moulds. With about 300 recognized phylogenetic species, *Fusarium* is one of the largest genera of fungi. Its complex phylogeny comprises 29 lineages including 20 species complexes and 9 single species [1]. The taxonomic delimitation of the genus is currently a matter of debate: a small generic concept that splits *Fusarium* in several genera (e.g., *Neocosmospora*) [2,3] and a wide generic concept that keeps *Fusarium* with all species complexes including the *Fusarium solani* species complex (syn. *Neocosmospora*) [4,5]. We use the latter concept, because the morphological recognition of *Fusarium* spp. is of great importance in diagnostics.

In nature, *Fusarium* spp. are present on plants, in the soil, air as well as water [6]. The majority of species, such as *F. verticillioides* and *F. graminearum*, are typically plant pathogens and therefore contribute to some of the greatest problems facing humanity, namely hunger and malnutrition. A common characteristic of *Fusarium* spp. is their pronounced capability to produce a wide range of mycotoxins, e.g., trichothecenes, including nivalenol, deoxynivalenol (also known as vomitoxin), fumonisin and zearalenone, all of which are ingested with food, and the consequences of which are largely underestimated by the human medical field [7].

From a medical perspective, *Fusarium* spp. are rather harmless environmental microbes that rarely cause human infections [6]. Opportunistic species are occasionally found as pathogens in patients with impaired immune systems, e.g., patients with leukemia, and can lead to a disseminated infection affecting practically all organs [7].

In addition, *Fusarium* spp. are a leading cause of fungal eye infections which have shown an increasing incidence in recent years [6,8]. Even keratitis outbreaks have been reported associated with the use of a certain brand of lens solutions [9,10]. In contrast to bacterial forms of keratitis, the course of Fusarium keratitis is usually more progressive [6,8] and the consequences generally more serious [8,11,12], not least because the antifungal therapy is less efficient, since these fungi are a priori resistant to many types of antimycotics [8,11,13]. There is evidence that these pathogens possess certain virulence factors that enable destruction of tissue barriers and progression [6,14]. Fungi from the *F. solani* species complex such as *F. falciforme*, *F. keratoplasticum*, *F. petroliphilum* are particularly common in cases of Fusarium keratitis besides *F. oxysporum*, *F. proliferatum* (out of the *F. fukikuroi* complex) and others [6,8,11].

We present a case in which the patient developed an infectious keratitis due to *F. tonkinense* (syn. *Cylindrocarpon tonkinense*, *Neocosmospora tonkinensis*) of the *F. solani* species complex. Despite intensive conservative and subsequently surgical therapy, the clinical course deteriorated rapidly with spreading of the infection into the anterior chamber and finally into the vitreous body.

## 2. Case Report

### 2.1. Clinical Presentation

**Figure 1:**
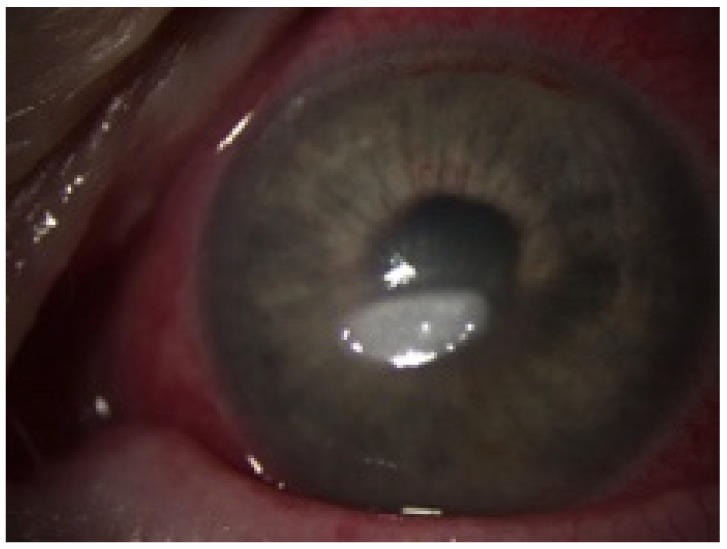
Biomicroscopic slit-lamp photography of the left eye upon inpatient admission. Clinical findings demonstrated a white, gelatinous paracentral corneal infiltrate (3.0 × 1.5 mm) and discrete peripheral infiltration (0.5 mm) at 10 o’clock.

A 58-year-old woman presented at the hospital with a corneal infiltrate that had been refractory to the outpatient treatment. According to the referring doctor, it had started with an edematous lesion approximately one week ago and was initially treated with topical antibiotics (ofloxacin) and steroids (dexamethasone). Acyclovir ointment was added because of the development of a presumed herpes-like ulcer, but without satisfying results. At the time when the patient was admitted to hospital, the cornea showed a white, gelatinous paracentral corneal infiltrate measuring approximately 3.0 × 1.5 mm and a discrete peripheral infiltration measuring about 0.5 mm (Figure 1). The infiltrate was surrounded by an epithelial edema and the endothelium had some dense whitish plaques. The conjunctiva showed a circularly moderate injection. The subsequent inflammation in the anterior chamber manifested itself with aqueous flare of 2+ and inflammatory cells of 1+. Fundus and vitreous body showed no ophthalmoscopic or sonographic evidence of inflammation.

The patient’s history did not reveal any evidence of a (micro)traumatic incident, contact lens use, or an ocular disease in the past. Systemically, the patient suffered from drug-treated arterial hypertension. There were no indications for a diabetic disease. Four years ago, the patient had undergone radiotherapy and chemotherapy because of a carcinoma of the lower jaw, in addition to a surgical tumour resection (up to now without recurrence). Additionally, she suffered from a (progressive) depressive disorder requiring medically supervised drug therapy.

**Figure 2:**
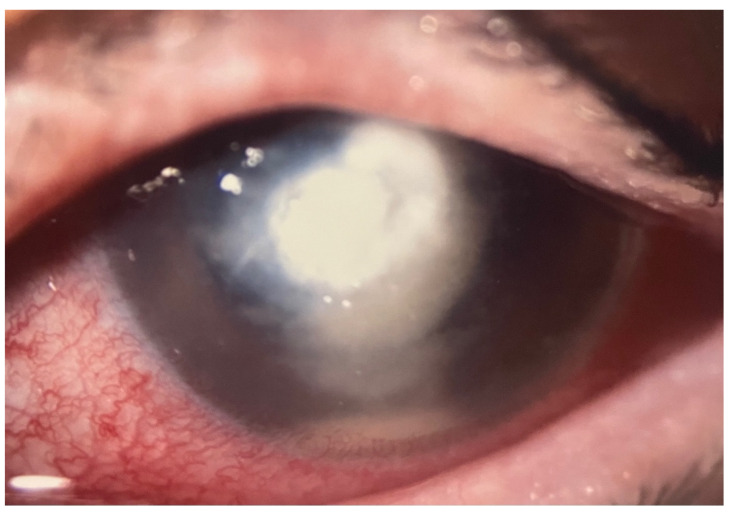
Biomicroscopic slit-lamp photography of the left eye before the first keratoplasty with significant expanded infiltration penetrating all corneal layers and intrusion in the anterior chamber.

Due to the initial clinical suspicion of a fungal infection at hospital admission, diagnostic corneal abrasions in the affected area were repeatedly performed. However, the microbiological findings did not show evidence of microorganisms (HSV-, VZV-PCR, bacterial growth, microscopic examination for acanthamoeba or fungi, respectively). Despite adjusting and intensifying topical therapy, using azithromycin and ganciclovir, and subsequently voriconazole (2%), the infiltrate began to spread extensively to all corneal layers and a white gossamer structure, attached to the posterior cornea, developed. Given the foudroyant progression (Figure 2) (and the continued lack of evidence of specific pathogens), a therapeutic penetrating keratoplasty (graft diameter of 8.0 mm) was performed with complete macroscopic removal of the lesion (8 weeks after the onset of the disease). During the first postoperative days, we observed an anterior segment almost without irritation and a clear graft and host cornea.

In the excised host cornea, *Fusarium* spp. was identified by culture for the first time. However, the isolate showed high MICs for most antifungals except of natamycin (Table 1).

**Figure 3:**
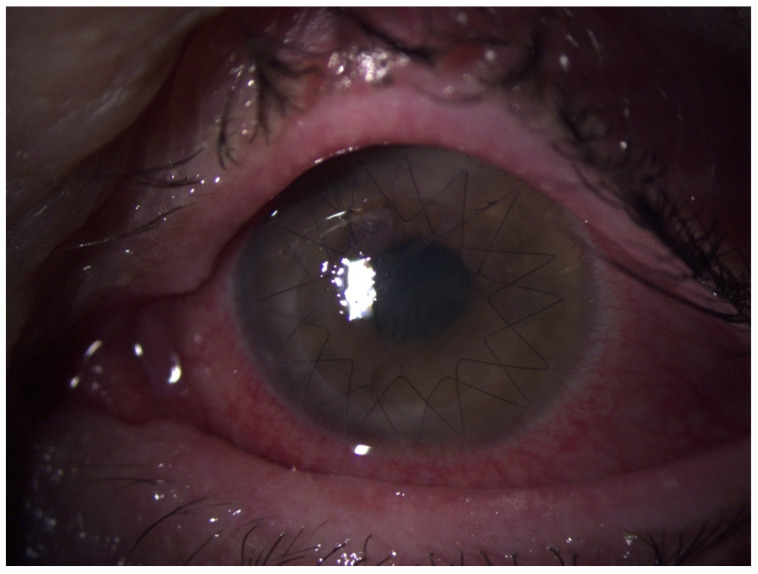
Biomicroscopic slit-lamp photography of the left eye one week after first keratoplasty with again incipient infiltration spreading from the remaining host cornea.

**Figure 4:**
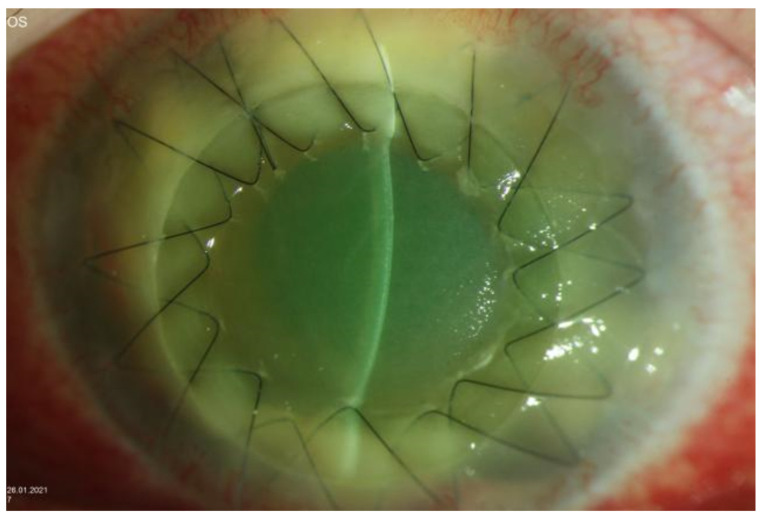
Biomicroscopic slit-lamp photography of the left eye 3 weeks after the first keratoplasty with extended circular infiltration of the host and graft cornea. The epithelial defect is due to repeated corneal abrasion to improve drug penetration.

Due to the development of a new corneal infiltrate starting from the remaining host cornea (Figure 3) rapidly spreading to the graft (Figure 4), the treatment was extended with topical natamycin and polyhexamethylene biguanide (at times applied hourly), systemic voriconazole (200 mg 2× daily) and terbinafine (250 mg 1× daily), as well as continuous intracameral and intravitreal injections of voriconazole (100 µg/0.1 mL) and amphotericin B (7.5 µg/0.1 mL) at intervals of several days. Voriconazole was also administered intrastromally in the peripheral cornea. Repeated microbiological examinations of samples collected from the anterior chamber and vitreous body provided no new findings.

Despite a short period of clinical improvement during the above-mentioned treatment, the overall course of the disease was further progressive and a large-diameter repeat keratoplasty (graft diameter of 13.0 mm, 32 interrupted sutures) was performed 1 month after the first keratoplasty.

**Figure 5:**
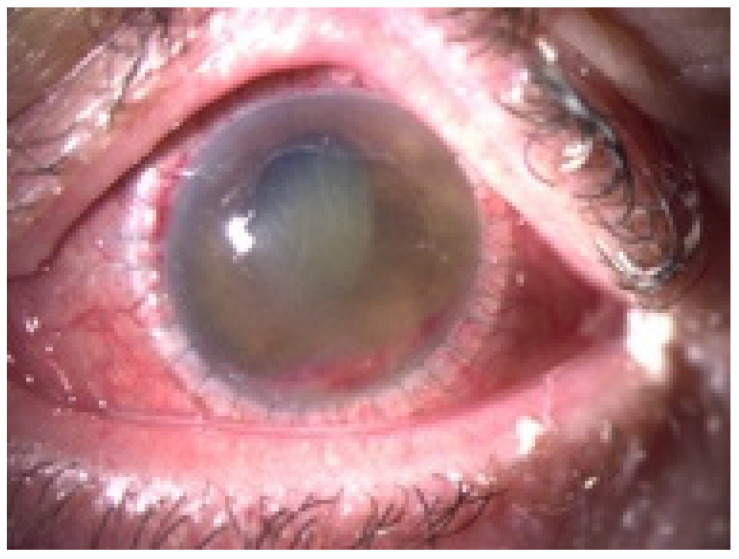
Biomicroscopic slit-lamp photography of the left eye one week after a large-diameter (13.0 mm) repeat keratoplasty with again white-viscous material in the inferior iridocorneal angle.

The histologic examination of the corneal explant (graft with adjacent host tissue) demonstrated that the focus of inflammation with fungal hyphae was mainly located in the posterior corneal stroma of the host cornea near the interface area. The corneal graft showed an acute ulcerative keratitis close to the graft margin. Following once again an only minimal-irritative anterior segment status and clear corneal transplant within the first postoperative days, white, viscous material sprawling from the iridocorneal angle (Figure 5) appeared and the corneal transplant and the anterior chamber developed a progressive diffuse infiltration.

**Figure 6:**
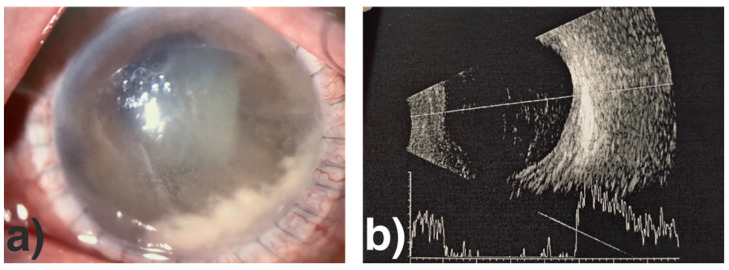
Biomicroscopic slit-lamp photography (**a**) and B-scan (**b**) of the left eye before enucleation with continuing progression in the anterior segment and beginning endophthalmitis.

Over the next weeks and months with continued intensive topic and systemic therapy as well as intracameral and intravitreal injections, the course of the disease stagnated indeed temporarily, but the overall status continued to deteriorate and, finally, the vitreous body became affected, too (Figure 6a,b).

With the continuing progression of the endophthalmitis (and at the patient’s urging) after several months of treatment with considerable mental and physical strain, we eventually decided to proceed with the ultima ratio of enucleation. This intervention and the subsequent course passed without further complications. Within a short period of time the conjunctiva of the eye socket was completely irritation-free and the patient could be fitted with a cosmetically pleasing prosthesis.

### 2.2. Histology

After the removal of specimen for microbiologic examinations (s. above), the enucleated eye was preserved at −70 °C and then immediately after thawed in 8% formalin solution.

**Figure 7:**
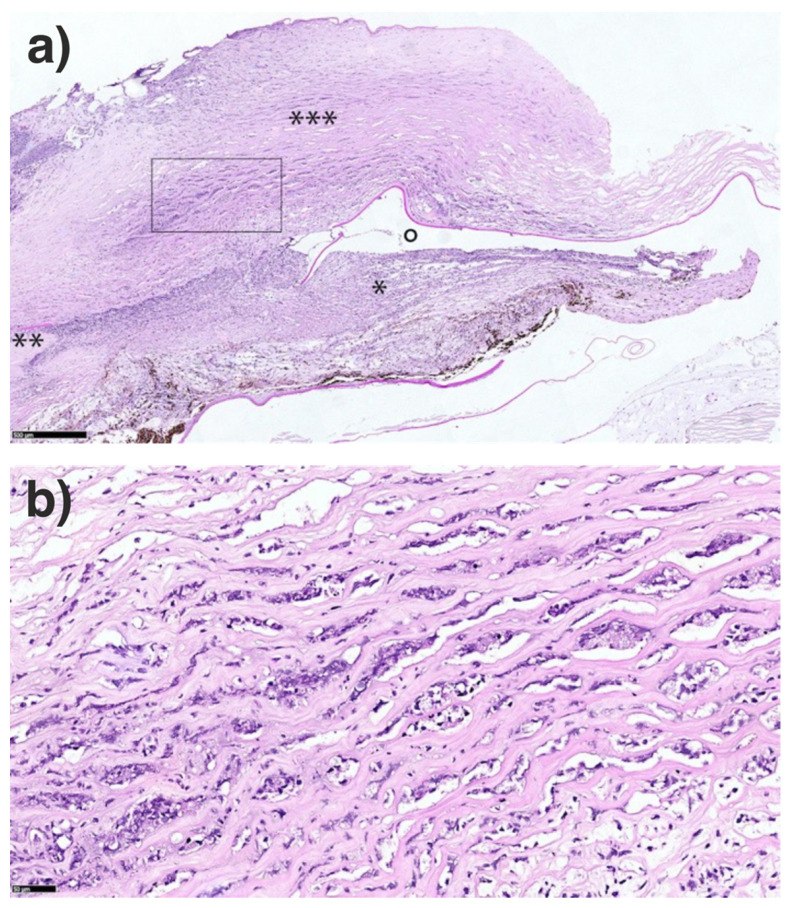
Histopathological examination of the anterior segment. (**a**) Necrotizing inflammatory reaction consisting of neutrophil granulocytes mainly in the cornea (***) and adjacent chamber angle (**), iris (*) and anterior chamber (°). Periodic acid Schiff stain, bar = 500 µm. (**b**) Enlarged area inside the square of figure a containing necrotic neutrophil granulocytes between the corneal stroma lamellae (Periodic acid Schiff stain, bar = 50 µm).

The histologic examination revealed a necrotizing inflammatory reaction consisting of neutrophil granulocytes mainly in the cornea and adjacent chamber angle, iris and anterior chamber (Figure 7a,b) but could not detect (neither with the Grocott Gomori stain nor with the PAS stain) fungal elements within the whole eye.

**Figure 8:**
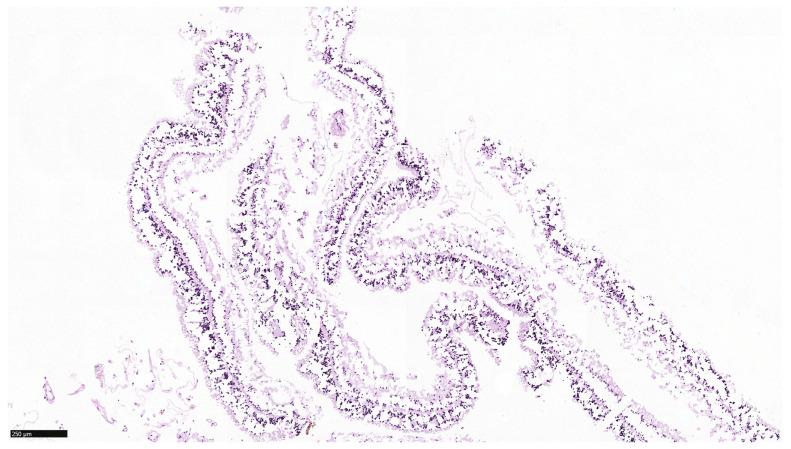
Artificially detached normal appearing retina with lytic changes, which rather represent a freeze-thawing artifact. The artificial detachment is due to pre-freezing manipulation to obtain microbiological samples (Periodic acid Schiff stain, bar = 250 µm).

The vitreous contained sparse neutrophil granulocytes, few unspecific lymphocytic infiltrates were found in the choroidea. There were lytic changes of the otherwise normal appearing retina, which rather represented a freeze-thawing artifact. The artificial detachment of the retina was due to pre-freezing manipulation to obtain microbiological samples (Figure 8).

### 2.3. Microbiology

No bacteria were identified using eubacterial PCR testing in the intraoperatively collected corneal sample during the first therapeutic keratoplasty. However, the panfungal PCR results were positive. Blood agar, chocolate blood agar, and Sabouraud agar were inoculated. After 3 days of incubation at 26 °C, a mould was isolated. No bacterial growth was identified on blood, chocolate or McConkey agar.

**Figure 9:**
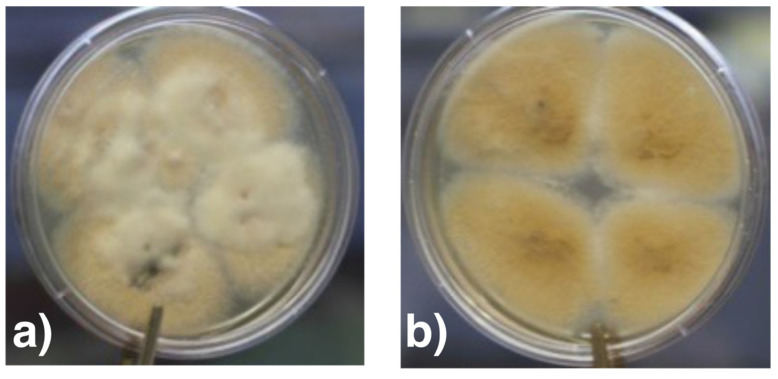
Colony morphology of isolated *Fusarium* sp. after 10 days on Sabouraud agar at 26 °C. (**a**) Front side: Fluffy colony (**b**) Reverse side: ochre-coloured pigment.

**Figure 10:**
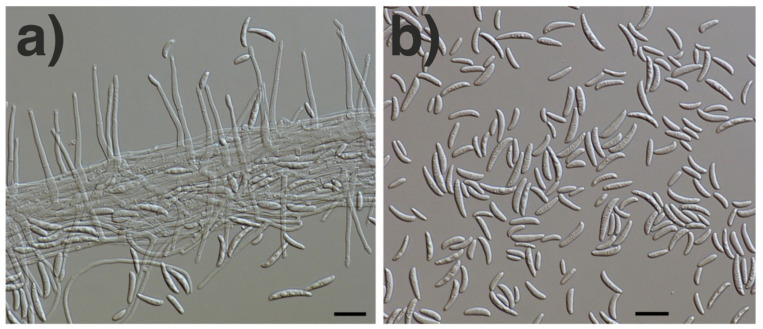
Micromorphology of *Fusarium tonkinense* (differential interference contrast microscopy). (**a**) Mycelial strand with conidiohores. (**b**) Curved microconidia. Scale bars = 20 µm.

Based on the colony morphology on Sabouraud agar (Figure 9a,b) and the micromorphological characteristics (Figure 10a,b), the fungus was classified as *Fusarium* spp.

Molecular identification via amplification/sequencing of the ITS (internal transcribed spacer) region and the translation elongation factor 1α (TEF) were performed as described previously [8]. The ITS and TEF sequences unambiguously identified the strain as *F. tonkinense*: the ITS sequence (GenBank accession number MZ707714) was 100% identical with that of the ex-type strain of *F. tonkinense* CBS 115.40 (GenBank accession number: NR_170733). No TEF sequence of the ex-type strain of *F. tonkinense* is available but the TEF sequence of NRZ-2021-193 (GenBank accession number: MZ703012) shows 100% similarity with strain IFM:JPN:62225 (GenBank accession number: LC177294) which is a reference strain of the phylogenetic species 9 of the *Fusarium solani* species complex (FSSC 9) that is now named *F. tonkinense* [15]. The strain is deposited in the Jena Microbial Resource Collection (JMRC): NRZ-2021-193.

Antifungal susceptibility testing was performed by microdilution using the EUCAST protocol [16]. The isolate showed high MICs for most antifungals except for natamycin which is considered to be effective up to MICs of 16 mg/L, as this concentration can be reached at the surface of the eye (Table 1) [17].

The determination of mycotoxin production was carried out in the culture supernatant of the strain in sabouraud broth for 16 days at 26 °C (analogue to the temperature of the eye surface) using ELISA (Immunolab RAPID ELISA: Fumonosin; R-Biopharm-RIDASCREEN: deoxynivalenol, zearalenone, T-2 Toxin). The patient’s strain clearly produced none of the tested mycotoxins in detectable quantities (Table 2).

Cultures from samples of the anterior chamber fluid and vitreous body from the enucleated eye were negative (neither bacteria nor fungi could be cultivated). The panfungal PCR also showed no signal.

## 3. Discussion

Fusarium keratitis is relatively common in tropical regions such as South India, and *Fusarium* spp. are among the leading causes of visual impairment and blindness [18]. In South India, infections are usually found in rural settings, so that it is quite common that agricultural workers in India often become infected after a corneal injury caused by plant or soil material [19]. Fungal keratomycosis in Germany is caused by several agents and among them *Fusarium* spp. as described in the German keratitis registry [20].

Species of the *Fusarium solani* species complex are the prevailing agents of Fusarium keratitis [8,21,22]. In Germany, the most common species isolated from keratomycoses are *F. petroliphilum*, *F. keratoplasticum* and *F. solani* [8].

*Fusarium tonkinense* was originally described as *Cylindrocarpon tonkinense* in 1939 [23]. In phylogenetic studies, the species was designated as phylogenetic species 9 of the *Fusarium solani* species complex. When it was recognized that sequences of the ex-type strain of *Cylindrocarpon tonkinense* fit in this clade, it was renamed *Neocosmospora tonkinensis* [2] and later transferred to *Fusarium tonkinense* [5]. Using these different names, this species has been reported to cause keratitis in several countries including Colombia [24], Germany [8,11], Japan [15], UK [25] and USA [26].

The predisposing factors are numerous but often remain unclear in individual cases. The principal risk factors are the use of contact lenses and trauma or operative intervention, damage to the cornea, or blocked tear ducts [14]. In Germany, the majority of affected patients are otherwise healthy women of approximately 50 years of age [11]. Indeed, none of the common predisposing factors were identified in the case presented here.

The infection generally does not remain limited to only the cornea but rather breaks through the anatomical barrier, namely Descemet’s membrane, which allows the pathogen to penetrate into the eye causing endophthalmitis. With the help of mannoproteins on their surface, *Fusarium* spp. are able to adhere to laminins, fibronectins and collagens on the cornea and propagate at the given temperature [14]. Furthermore, *Fusarium* spp. are able to use their genetic repertoire to create a biofilm covering the cornea. This protects them against numerous non-specific immune mechanisms and against topical antifungals [6]. Once they produce large amounts of proteases, phospholipase, and cytotoxic peptides [27], they can destroy the antimicrobial oligopeptides of the non-specific defences (such as lysozyme and defensins) and trigger the development of corneal ulceration causing penetration and enabling the invasion of the anterior chamber [6,14].

As soon as this hurdle has been overcome, severe inflammatory reactions are triggered because the pathogen-associated microbial patterns (PAMPs) of the fungi, such as glucan (large amounts of which are contained in the cell walls), activate the pattern recognition receptors (PRRs) of the host cells, especially dectin 1 and CD36. This increases the levels of proinflammatory cytokines IL-1, IL-6, IL-8, IL-17, IL-23 and IFN-γ in the aqueous humour significantly [14].

In such an eye with an intensive inflammatory reaction, anatomical alterations may develop which in turn restricts the outflow of aqueous humour, causing increased intraocular pressure and possibly leading to therapy-resistant glaucoma [6]. In the isolate NRZ-2021-193 particular virulence factors are not assessed. Whereas in principle, destructive, cytotoxic and immunotoxic mycotoxins, especially trichothecenes [7] acting as virulence factors [28] or as pathogenicity factors [29], might support the pathogenetic development of the fusarioses [6], mycotoxin production could, however, not be detected in the strain NRZ -2021-193 (Table 2), at least under the given, i.e., rather growth promoting, in vitro nutritive conditions [7]. This does not exclude entirely that under the local conditions on the eye and the hostile environment due the presence of defence mechanisms, such as lysozyme, mycotoxin production may have occurred anyway and possibly supported the progression. Another particular characteristic of *Fusarium* spp. is the development of adventitious spores within an infected tissue [30]. This phenomenon has also been described in a *Fusarium* strain isolated from keratitis [31]. The pathogenic role of this trait in keratitis remains obscure until now, but it could be responsible for clinical failure of antimycotic therapy and for persistence.

Manifestation in the deep corneal stroma is typical for *Fusarium* spp., which is why identification of the pathogen by corneal swabs or scrapings is often negative [32]. Especially for deep stromal manifestation, in vivo confocal microscopy can contribute to initiate a rapid empirical therapy by identifying the fungal elements (shown as hyperreflective parallel lines). In the later course, therapy can be adjusted according to closer pathogen identification. This therapeutic modality has a high sensitivity and may prevent disease progression with serious outcome as a supplement to other diagnostics (microscopic examination, culture, polymerase chain reaction) [33,34].

Fatal progression—even after large-diameter keratoplasty [35]—is partly explained by the fact that such an infection is difficult to treat with drugs, since *Fusarium* spp. are inherently resistant to many antifungals [11,13,36,37,38] and even to the polyene amphotericin B disposing MIC_90_ levels of >1 mg/L [13]. According to a study by Lalitha et al., natamycin could be considered as effective as voriconazole for *Fusarium* species, provided corneal penetration is not an issue [17]. Indeed, the concentration in the 5% solution is far beyond the MIC, namely 50.000 mg/L. In antifungal susceptibility testing based on the EUCAST protocol, all tested species, except for some strains of *F. solani*, showed natamycin MIC ≤ 8 mg/L [8] while in tests performed according to the CLSI protocol only 65% of the species showed natamycin MICs ≤ 16 mg/L [18]. One explanation for the relatively good activity could be a particular mechanism of action of natamycin against *Fusarium* spp. [39,40]. A prolonged topical therapy using natamycin (5%) is reported as an option to prevent progression or recurrence [41], using increased concentrations hourly or in even shorter treatment intervals and trying to achieve an effective local concentration through topical application. Since the half-life of topically administered drugs is only about 4 min [42], it is recommended that the eye lids should remain closed for about two minutes, so that definitely higher amounts of the agents will persist in the tear film than compared to active movements of the lids [43]. In addition, the naso-lacrimal ducts should be compressed mechanically with the patient’s fingers so that the drug cannot evade through this pathway [44]. High-frequency application is also important. However, natamycin has a high molecular weight and is therefore unable to penetrate the corneal epithelium or even into deeper compartments of the eye [13,32]. Thus, in order to increase the probability of therapeutic success by promoting the access of the drug into the infected eye compartment, an abrasion of the epithelium is recommended. In the future, luliconazole, an imidazole derivative, might provide a highly effective treatment method against *Fusarium* spp. [45]. This may also be held true for fosmanopegix, a first-in-class prodrug of manogepix. This drug active against *Fusarium* spp. interfering specifically with the synthesis of a fungal protein of the cell wall, namely mannoprotein representing an essential constituent, is in the pipeline for ophthalmologic use. It is well tolerated after oral as well as intravenous application because of the selective mechanism of action [46].

Intrastromal administration of voriconazole might be an effective way of providing higher concentrations of the drug especially when there is a risk of conreal mel and perforation. Indeed, intrastromal injection of (0.05–0.1 mL) aided the resolution of various fungal infections [47].

For intraocular infections, voriconazole [48] and/or amphotericin B intraocular injection may be required in an attempt to achieve a concentration above the MIC with the aim of combatting the otherwise resistant pathogens (Table 1) [32].

Voriconazole is actually the most important antifungal, which in addition to locally applied drugs, can be given intravenously. This therapy should be taken into consideration irrespective of the in vitro susceptibility, since good clinical response in hematologic patients could be observed even when administered in cases with resistant pathogens and vice versa [49]. Another option could be terbinafine, although the final clinical value of this drug in clinical cases of fungal keratitis caused by the *Fusarium* spp. remains still to be elucidated. Whereas this antimycotic showed good in vitro activity at least against some *Fusarium* spp. [13,32,50], high MIC values are reported, too [19]. Indeed, the tested strain of *F. solani* displayed high MIC values (Table 1).

An in-vitro determination of an antimycogram of *Fusarium* spp. isolated from the cornea is not routinely done and indeed not really necessary and the predictive value for outcome is not stringent. First, because the interpretation of the results is difficult and even arbitrary, since standardized, authoritative breakpoints are not yet available. Second, because for topically applied antimicrobials in general this classification in “susceptible” or “resistant” is dispensable, since the concentrations in eye drops can definitely exceed the measured MIC values.

Despite clinical progression, histology did not reveal any fungi, neither in GMS- nor in PAS-staining, indicating microbiological therapeutical success. Nevertheless, acute necrotizing inflammation was present at the time of enucleation which could be an overreaction of immune response, even after *Fusarium* as the initial cause was eliminated as described in the literature [12] raising the question of additional anti-inflammatory treatment at this stage of the disease. Repeated antifungal applications did not cause any retinal damage, which was reported especially for the pore-forming amphotericin B. This substance also binds to cholesterol in human cells, but with a 1000-fold lower affinity than to ergosterol in fungal cells. Combinations with terbinafine, which demonstrated good in-vitro activity against plant pathogenic *Fusarium*, have been mentioned sporadically in fungal keratitis [34,51] when administered orally and topically, although there is not yet sufficient evidence for their clinical efficacy.

## 4. Conclusions

The case illustrates the often extremely difficult treatment of ocular fungal infections. Despite the broad spectrum of available antifungal agents, even their highly intensive and combined application does not lead to satisfying treatment results in some cases. The risk of drug-induced tissue damage should not be underestimated, especially in case of high-dose and long-term use. It is known that *Fusarium* spp. are resistant to many of the available antifungal agents. The *F. tonkinese* strain detected only showed a response to natamycin with an effective concentration achievable in the eye. In clinical use, however, natamycin, as well as voriconazole, terbinafine, amphotericin B or polyhexanide (often effective substances in other cases) remained without significant success. Affection of the vitreous body was presumably prevented for a long time by the preserved iris-lens diaphragm and frequent intravitreal drug administration. Drug-induced retinal damages were not found in the histological examination. For refractory infectious keratitis, surgical intervention should be performed as early as possible to prevent anterior chamber involvement. Retrospectively, a large diameter with single interrupted sutures should have been chosen for the first keratoplasty. In general, topical steroids should be avoided before and immediately (at least for 10 days) after corneal transplantation. In case of recurrence of anterior chamber inflammation, frequent irrigations with drug administration (voriconazole 2% and amphotericin B 0.15%) should be performed. However, in the beginning, no etiologic agent had been identified, especially no fungi, and a keratoplasty including the limbal area involves the potential of severe complications. An astonishing fact is that fungi were not detectable in the enucleated eye (neither histologically nor microbiologically). Obviously, the aggressive therapy finally led to an elimination, but it did not stop the persistence of the inflammatory response. In this context, the (time-delayed) use of steroids should have been be considered earlier.

Although the outcome in this case was unfortunately frustrating, a rapid diagnosis and a prompt therapy initiation (preferably based on a resistogram) are crucial, especially in the case of mycotic infections. New active agents, which are currently undergoing clinical trials, also offer the prospect of further optimized therapeutic approaches in the future.

## Figures and Tables

**Table 1:** Antifungal susceptibility testing results for *F. tonkinense* NRZ-2021-193.

Antifungal	MIC	Assessment *
Amphotericin B	2 mg/L	presumably resistant
Natamycin	4 mg/L	presumably susceptible
Caspofungin	>8 mg/L	presumably resistant
Anidulafungin	>8 mg/L	presumably resistant
Itraconazol	>8 mg/L	presumably resistant
Isavuconazol	>8 mg/L	presumably resistant
Posaconazol	>8 mg/L	presumably resistant
Voriconazol	8 mg/L	presumably resistant
Terbinafin	>32 mg/L	presumably resistant

* Since official breakpoints are not given (neither by EUCAST nor by CLSI) the judgement is more or less arbitrary.

**Table 2:** Mycotoxin production (DL: Detection limit).

Mycotoxin	Group	Amount
Deoxynivalenol (also known as vomitoxin)	Trichothecene	<20 g/L (DL)
T2 Toxin (also known as fusariotoxin)	Trichothecene	<18 µg/L (DL)
Fumonisin B1	Fumonisin	<0.05 mg/L (DL)
Zearalenone	Zearalenon	<2 µg/L (DL)

## Data Availability

The data presented in this study are available on request from the corresponding author.
